# Is it necessary to screen for celiac disease in adult idiopathic osteoporosis? 

**Published:** 2015

**Authors:** Bijan Shahbazkhani, Najmeh Aletaha, Ahmad khonche, Benyamin Farahvash, Reza Malekzadeh

**Affiliations:** 1 *Digestive Disease Research Institute, Tehran University of Medical Sciences, Tehran, Iran*; 2 *Department of Gastroenterology, Tehran University of Medical Sciences, Tehran, Iran*; 3 *Baqiyatallah University of Medical Sciences, Tehran, Iran*; 4 *Boston University School of Medicine, USA*

**Keywords:** Celiac Disease, Osteoporosis, IgA anti-tissue transglutaminas

## Abstract

**Aim:**

the aim of this study was to investigate the necessity of screening for celiac disease in idiopathic osteoporotic patients.

**Background:**

Osteopenia and osteoporosis are well-known and prevalent complications of celiac disease. However, the relative prevalence of celiac disease among osteoporotic populations is not known, and the benefit of screening for celiac disease among the osteoporotic population remains controversial.

**Patients and methods:**

We evaluated a total of 560 individuals, 460 with osteoporosis and 100 healthy subjects, from the rheumatology clinic in Imam Khomeini and Shariati hospital by IgA anti-tissue transglutaminase (anti-tTG) for celiac disease. Then individuals with positive serologic test underwent upper GI Endoscopy & 2nd part duodenum biopsies. The clinical findings were evaluated in both groups and were compared with each other.

**Results:**

Five (1.08%) of 460 patients with osteoporosis and 1 (1%) of 100 subjects without osteoporosis had celiac disease by positive serologic & pathology results. Three patients with positive serology & pathology results were female. All patients in osteoporotic group had at least one other symptom of celiac disease. Two of them had anemia and others had chronic abdominal pain, recurrent oral aphtous lesion & chronic bloating.

**Conclusion:**

In the present study, the prevalence of celiac disease in osteoporotic patients is not high enough to justify recommendation for serologic screening of celiac disease in all patients with idiopathic osteoporosis; but in osteoporotic patients with other celiac or gastrointestinal symptoms and signs, for example iron deficiency anemia, chronic dyspepsia and bloating, constipation or diarrhea and recurrent aphtous lesions, it is necessary to evaluate for celiac disease.

## Introduction

 Celiac disease (CD) has been assumed to be a gastrointestinal (GI) problem (1), but it has remarkable extra GI manifestations, which may deviate physicians from correctly diagnosing the disease. Celiac disease has been reported with single manifestations such as anemia, infertility, and recurrent aphtous stomatitis. On the other hand, celiac disease usually causes complications such as malabsorption. (2-4) Osteopenia and osteoporosis are well-known consequences of celiac disease (5). Osteopenia and osteoporosis are the common complications of celiac disease, which increases with age. (6-8). Adults with recently diagnosed celiac disease have osteopenia or osteoporosis diagnosed by bone absorptiometry (9), and treatment of these subjects with a gluten-free diet increases their BMD. (10-14). Interestingly, low bone mineral density (BMD) might be the early or preceding symptom of celiac disease, and bone mineral density may be even lower in clinically silent CD patients than in symptomatic celiac patients (6, 15-16). There are conflicting data for the presence of celiac disease among the idiopathic osteoporotic patients. (17-25).

**Table 1:** Demographic features, of the case and healthy group

	**Osteoporotic (case group; n = 460)**	**Non-osteoporotic** **(healthy group; n =100)**	**P-value**	**Normal values**
Age (years)	39± 13.21	37± 13.12	0.927	
Body mass index (kg/m2)	28.22 ± 3.5	28.32 ± 3.3	0.711	
Lumbar spine BMD (T-score)	-2.87 ± 0.70	-2.64 ± 0.62	0.183	
ALk.P (U/l)	215.23 ± 63.20	226.79 ± 68.31	0.330	98–279
Calcium (mg/dl)	9.56 ± 0.54	9.61 ± 0.52	0.589	8.5–10.5
Phosphorus (mg/dl)	3.67 ± 0.53	3.63 ± 0.51	0.980	2.7–4.5
PTH (pg/ml)	39.19 ± 21.13	40.20 ± 21.85	0.769	10–70
25-OH-vitaminD (ng/ml)	29.31 ± 15.36	29.23 ± 11.48	0.987	10–50

Prevalence of celiac disease among Iranian osteoporotic patients is not well studied (26, 27). Therefore, we conducted a case control study to investigate if we need to screen celiac disease in osteoporotic patients. 

## Patients and Methods

This case-control study was conducted from January 2009 until December 2010. Demographic data of these subjects mentioned at table 1. During this period we enrolled subjects who were referred from rheumatology clinic of Shariati and Imam Khomeini hospital and rheumatology clinic of Shariati hospital to the bone densitometry center and fulfilled our inclusion criteria. Cases included individuals with a diagnosis of osteoporosis and healthy individuals acted as controls. Bone mineral density (BMD) was measured in all patients by dual energy x-ray absorptiometry at the lumbar spine (antero-posterior projection of L1–L4). The World Health Organization definitions were used to define osteoporosis. According to the WHO criteria, osteoporosis is defined as a BMD 2.5 standard deviations or more below the average value for young healthy women (a t-score of <-2.5 SD)(28). Exclusion criteria for this study were diseases well known to affect bone metabolism (thyroid disease, parathyroid disease, renal disease, liver disease, and malignancy), individuals with known celiac disease and any medication known to affect bone turnover such as glucocorticoids and anticonvulsant drugs. After getting written informed consent from the patients, two researchers filled a structured questionnaire that included closed questions. Then, 5 ml venous blood of each patient was obtained in clotting tubes. The samples were sent to the laboratory of Digestive Disease Research Center for centrifuging and extracting serum samples for total IgA and, IgA anti tissue transglutaminas (IgA anti TTG) test. The tests were done using kit with cut off point of 7 mu/ml. Individuals testing positive for IgA anti TTG were offered an upper GI endoscopy with 4 random biopsies of the distal duodenum. Biopsy samples classified according to Marsh staging. 

## Results

Four hundred sixty subjects with a mean age (± SD) of 39±13.21 years, who met our inclusion criteria of osteoporosis, were enrolled in our study. We also enrolled 100 healthy subjects with a mean (± SD) age of 37±13.12 years. Among the osteoporotic patients, 250 (54.34%) were female of which 140 (56%) were postmenopausal. Sixty-four (64%) of healthy subjects were female. The mean levels of serum calcium, phosphorus, alkaline phosphatase, PTH, 25 (OH) vitamin D were similar in both groups and also in celiac patients (P > 0.05) (table 1).

The results of IgA anti-TTG test for 6 subjects were positive in 5 of the cases and 1 of the control group. These positive IgA anti-TTG patients underwent upper GI endoscopy. Four quadrant biopsies from 2nd part of duodenum were taken. Four cases had Marsh 2 and 2 of them had Marsh 3A in pathology biopsy. Among the positive serology and biopsy test cases, 3 (60%) were female and 2 (40%) were male. In healthy group there was only one female patient, which had positive IgA anti-TTG test. Two celiac patients of osteoporotic group were postmenopausal and the only patient with celiac disease from healthy group was postmenopausal. All celiac patients in case group had at least one other manifestation including anemia (IDA), recurrent abdominal pain, chronic bloating, and recurrent oral aphtous ulcer (table 2).

## Discussion

The present study evaluated celiac disease by serology and tissue diagnosis among patients with idiopathic osteoporosis. 

Celiac disease is often associated with low bone density and patients with celiac disease have an increased fracture risk, a hazard ratio of 1.43 or 43% increased risk when compared to age-matched healthy populations (29, 30). 

**Table 2:** Demographic features of the celiac patient in patient and healthy group

Osteoporosis	sex	age	Anti-TTG	Other Manifestations
Yes	F	37	18.3	Anemia (IDA)
Yes	F	32	24.6	Anemia (IDA) and constipation
Yes	M	57	23.7	Chronic bloating
Yes	F	49	29.1	Recurrent abdominal pain
Yes	M	53	22.9	Recurrent oral aphtous lesion
No	F	52	17.3	No symptoms

CD affects bone mineral metabolism by multiple mechanisms. The two main mechanisms by which celiac disease causes osteoporosis are systemic inflammation and intestinal malabsorption. Malabsorption cause general malnutrition, decreased bioavailability of calcium and vitamin D, lower body mass index (BMI) and reduced muscle mass (31-33). Vitamin D deficiency and low calcium concentrations are presented in 30%-60% of these patients (32). Secondary hyperparathyroidism is often present due to hypovitaminosis D, and lead to increased bone turnover (31, 35-37).

Inflammation modulates bone resorption mainly by two mechanisms. Firstly, pro-inflammatory cytokines have a final common mediator of osteoclast function: receptor activator of nuclear factor-B (RANK) and its functional ligand (RANK-L), also known as TRANCE (TNF-related activation induced cytokine) (35, 37). Gut inflammation also has a direct inhibitory effect on the usual inhibitor of this pathway, osteoprotegrin (OPG) (36, 37). Through this RANK/RANK-L/OPG pathway gut inflammation can have a direct negative effect on bone mineral density (BMD) that is independent of vitamin D absorption and could explain the degree of low BMD out of proportion to PTH and vitamin D levels (33-35). Secondly, osteoclastogenesis can be regulated through the modulation of macrophage colony stimulating factor. Osteoprotegerin autoantibodies may contribute to the pathogenesis of osteoporosis in celiac disease (39).

In many patients, extra gastrointestinal symptoms are the presenting complaint and should prompt the consideration of serologic testing. Metabolic bone disease is common in celiac disease and can occur in patients without gastrointestinal symptoms (6, 15-16). Iranian & other studies showed the prevalence of osteoporosis among patients with known celiac disease is higher than the general population (9, 28, 40-43).

Although there has been agreement that osteoporosis is a common manifestation of celiac disease, but data regarding the prevalence of CD in the patients with osteoporosis are conflicting. 

In this study, we planned to perform a case control study and serological screening for CD among osteoporotic patients (men & both premenopausal and postmenopausal women) and non-osteoporotic individuals. Celiac disease diagnosed in 6 subjects, 5 from cases (1.08%) and 1 from controls (1%). Our results showed the prevalence of celiac disease in idiopathic osteoporosis is not high enough to justify recommendation for serologic screening of celiac disease. 

The result of a case control study by Khoshnood et al. including 100 patients with osteoporosis and 100 healthy participants without osteoporosis, showed the higher prevalence of celiac disease in osteoporotic patients compared to healthy participants. (25). Armagan et al. studied 89 premenopausal women with idiopathic osteoporosis. Nine of 89 patients (10.11%) had celiac disease (positive for IgA endomyseal antibody). An increased prevalence of celiac disease in patients with idiopathic osteoporosis compared to the general population was suggested in this study. The lack of intestinal biopsy is the main limitation of this study (21). However, some studies fail to support an increase in the prevalence of celiac disease among patients with osteoporosis. Kavuncu et al. studied 192 postmenopausal women with low BMD, only one (0.5%) was positive for both IgA-AGA and IgA-EMA tests. Prevalence of CD in their patients (0.5%) did not differ from the prevalence of CD in normal healthy population (0.3–1%) (24). Isabelle Legroux-Ge´rot et al. studied 140 patients (133 postmenopausal women and 7 men) aged 40-75 years with primary osteoporosis diagnosed by absorptiometry. They measured IgG and IgA antigliadin antibodies. Patients with positive antigliadin antibody tests were tested for antitransglutaminase antibodies. They found no excess risk of celiac disease in their cohort of patients with osteoporosis (23). Stenson et al. evaluated 840 individuals, 266 with and 574 without osteoporosis, from the Washington University Bone clinic by serologic screening for celiac disease. Individuals with positive serologic test were offered endoscopic intestinal biopsy to confirm the diagnosis of celiac disease. The prevalence of celiac disease among osteoporotic individuals (3.4%) was much higher than that among non-osteoporotic individuals (0.2%) (34). But in our study we investigated men and premenopausal and postmenopausal women with low BMD and we found that the prevalence of CD in osteoporotic patient (1.03%) did not differ from the prevalence of CD in normal healthy population (1%). The same result was found in Gonzalez et al. study. They evaluated 127 postmenopausal patients (mean age: 68 years; range: 50-82 years) with osteoporosis. The observed prevalence of CD in this group was compared to that observed in a group of 747 women recruited for a population-based study. The screening algorithm used to diagnose CD was based on a 3-level screening using type IgA and IgG antigliadin antibodies (AGA) in all the patients (1st level) followed by antiendomysial antibodies (EmA) and total IgA (2nd level) of samples testing positive, and intestinal biopsy of positive cases (3rd level). There was no significant difference between the two groups (44). Also Laadhar et al. (45) found that the prevalence of CD in postmenopausal osteoporotic women was not statistically different from the prevalence of CD in general population.

The most important finding of our study is celiac disease in osteoporotic patients does not differ significantly from general population. Therefore, screening for CD seems to be not necessary in all osteoporotic patients, but it is reasonable to evaluate celiac disease in osteoporotic patients with other celiac symptoms and signs.
